# Challenges in Antimicrobial Treatment and Antimicrobial Stewardship in Hospital-Acquired Infections in Adult Burn Patients

**DOI:** 10.3390/ebj7020035

**Published:** 2026-06-10

**Authors:** Gianpiero Tebano, Caterina Convertino, Luigi Raumer, Rossella Sgarzani, Davide Melandri, Francesco Cristini

**Affiliations:** 1 Department of Medical and Surgical Sciences (DIMEC), Alma Mater Studiorum-University of Bologna, 40126 Bologna, Italy; rossella.sgarzani2@unibo.it (R.S.);; 2 Infectious Diseases Unit, Forlì and Cesena Hospitals, AUSL Romagna, 47121 Forlì and Cesena, Italy; 3 Infectious Diseases Unit, Taranto Hospital, 74121 Taranto, Italy; 4 Dermatology Unit and Burn Center, Cesena Hospital, AUSL Romagna, 47521 Cesena, Italy

**Keywords:** burns, bloodstream infections, wound infections, ventilator-associated pneumoniae, pharmacokinetic/pharmacodynamics, intensive care unit, antimicrobial stewardship

## Abstract

**Background**: Hospital-acquired infections (HAIs) represent the most significant complications in patients hospitalized for severe burn injuries, after the immediate post-burn resuscitation phase, and are associated with substantial morbidity and mortality. **Methods**: This is a narrative review. Evidence was extracted mainly with an in-depth search of MEDLINE, focusing on guidelines, randomized controlled trials, and relevant observational studies published in the last 25 years. The reference lists of the most relevant publications were screened to retrieve additional relevant information. **Results**: Wound infections, bloodstream infections, pneumonia, and urinary tract infections account for the majority of infectious complications. Their diagnosis can be challenging, particularly in the context of wound infections and sepsis. Burn severity and the resulting disruption of tissue and organ homeostasis can alter the pharmacokinetic and pharmacodynamic (PK/PD) properties of antibiotics, rendering standard dosing and administration strategies inadequate. Higher doses, prolonged or continuous infusions, and therapeutic drug monitoring may be required to optimize antibiotic exposure. The emergence of multidrug-resistant (MDR) pathogens (particularly MDR Gram-negative bacilli) has been widely reported across diverse epidemiological settings and occurs frequently in patients with prolonged hospitalization, further complicating treatment. As a result, the use of broad-spectrum antibiotics is substantial, both for empirical therapy and for targeted treatment. Although antimicrobial stewardship programs can promote more appropriate antibiotic use, evidence on how to effectively implement these strategies in Burn Intensive Care Units remains limited. **Conclusions**: HAIs in burn patients represent a highly complex clinical scenario. Clinical severity is often significant, diagnosis can be challenging, and MDR pathogens are very prevalent, with high consumption of broad-spectrum antibiotics. Moreover, PK/PD properties of antibiotics can be altered. Antimicrobial stewardship can promote appropriate antimicrobial use, but implementation in this setting has not been adequately studied. Close multidisciplinary collaboration between burn specialists and infectious diseases physicians is essential to ensure effective patient management.

## 1. Introduction

Burns are complex traumatic injuries caused by physical, chemical, electrical, and radiation agents, with thermal burns being the most common [1]. Burn injuries represent a major global public health problem, accounting for substantial mortality, morbidity, permanent disability, and a significant economic burden for healthcare systems. According to the World Health Organization (WHO), burn injuries cause an estimated 180,000 deaths annually. Most of these deaths occur in low- and middle-income countries, nearly two-thirds in the Africa and Southeast Asia WHO regions [1].

Thanks to the implementation of more effective prevention and treatment strategies, burn-related morbidity and mortality have decreased over recent decades, particularly in high-income countries, where specialized Burn Units are widely available [1,2]. To optimize treatment outcomes, the American Burn Association (ABA) has established specific criteria for referral to Burn Units; these include: full thickness burns and partial thickness ≥ 10% of total body surface area (TBSA); any partial or full thickness burn involving the face, hands, genitalia, feet, perineum, or joints; the presence of concomitant traumatic injury or suspected inhalation injury; pediatric patients; and chemical and electrical injuries [3]. In Burn Units, hospital-acquired infections (HAIs) are the most common complication and the main cause of death in patients surviving to the immediate post-traumatic resuscitation phase [4,5]. After the first 72 h, HAIs are estimated to account for approximately half of deaths in hospitalized burn patients, with this proportion potentially increasing up to 75%, in contexts with high prevalence of multidrug-resistant pathogens (MDR) [6,7,8].

Antibiotic treatment of burn patients is challenging for several reasons. First, the pharmacokinetics of antibiotics in burn patients can be significantly different compared to other patient populations, and they may change dynamically during the hospital stay [9]. Second, due to the high incidence and severity of HAIs in this setting [10], burn patients are frequently exposed to prolonged broad-spectrum antibiotics, leading to a high prevalence of infections caused by MDR pathogens. This has a direct impact on prognosis, since MDR infections in burn patients have been associated with a higher likelihood of progression to sepsis and need for mechanical ventilation (MV); a greater number of surgical procedures; longer duration of hospitalization and antibiotic therapy [6,11]. In this context, antimicrobial stewardship interventions are essential to preserve antibiotic efficacy at the patient and population level.

In this review, we summarize relevant information on the epidemiology, diagnostic challenges, antibiotic treatment, and antimicrobial stewardship aspects of HAIs in burn patients.

## 2. Methods

This is a narrative review. We screened the literature focusing on infectious complications in burn patients, including relevant papers and guidelines, particularly those published in the last 25 years. The search was mainly conducted on MEDLINE. The main search strings are listed in Table 1. The reference lists of the most relevant papers and documents were screened to retrieve additional relevant sources of information.

Although there were no strict exclusion criteria in the selection of screened papers, due to the non-systematic nature of this review, we focused on clinical studies involving humans, published after 2000 (preferably after 2010) in English, French, or Italian. We first searched for official guidelines or other types of guidance documents and for randomized controlled trials. Where evidence was not available from this type of publication, we also searched for relevant observational studies. Multidrug-resistant pathogens were defined according to the consensus definition by Magiorakos et al. [12].

## 3. Prevalence and Risk Factors of Hospital-Acquired Infections in Burn Units

The risk of burn injuries is related to low socioeconomic status [1,13]. Relevant risk factors for burns are male gender, younger age, poverty and overcrowding with poor safety measures, use of kerosene as fuel in households, work exposure to burning agents and high-voltage electricity, wearing flammable clothing, physical and cognitive disabilities, alcohol abuse, and smoking [1,14,15].

Severely burned patients have long hospital stays, a need for intensive healthcare support, and frequent invasive procedures, resulting in a high incidence of hospital-acquired infectious complications [4]. The prevalence and severity of HAIs have been strongly associated with the severity of burn injury, particularly with third-degree lesions and when burns involve more than 20% of the TBSA [8,16]. Numerous other factors have been associated with the onset of HAIs. Some of these factors are specific to the burned patient population, such as the presence of extensive tissue necrosis; burn mechanisms (flames); and the presence of inhalation injury. Other risk factors for HAIs are those encountered in other critically ill patients, i.e., prolonged hospitalization and use of invasive devices (e.g., MV, vascular catheters, urinary catheters) [16,17,18].

The main risk factor for the development of HAIs sustained by MDR pathogens is the length of hospital stay, which is influenced by the severity of burn injury and the presence of inhalation injury [6,19]. Other main risk factors for MDR pathogens, as in other settings, are prolonged exposure to broad-spectrum antibiotics, MV, and the presence of other invasive devices, clinical severity, and recent hospitalization [7,19,20,21,22,23,24,25].

Overall, according to the 2016 report of the National Burn Repository (NBR) [26], HAIs represent 7 of the 10 most frequent complications in burn patients. However, the prevalence of HAIs varies across different studies, as it is influenced by the characteristics of the included patients, particularly burn severity. In a large study by Strassle et al. including 4426 patients, HAIs were identified in 8% of patients, but patients with burns involving more than 20% of TBSA had a 10-fold higher risk [16]. In a study conducted at a regional Burn Center in Italy, Corcione et al. reported a higher prevalence of HAIs, ranging from 27% after 1 week of hospitalization, up to 44% after 4 weeks [17]. Tedesco et al. reported that sepsis was diagnosed in 20% of 1465 patients hospitalized in two burn centers in Canada [27]. Pneumonia (including VAP), urinary tract infections (UTIs), wound infections, and bloodstream infections (BSIs) are the most encountered HAIs [28]. Their prevalence can vary according to the study setting and study design [16,29,30].

## 4. Clinical and Diagnostic Peculiarities of Principal Hospital-Acquired Infections in Burn Units

### 4.1. Sepsis and Bloodstream Infections

Severe burn injuries determine a prototypical systemic inflammatory response syndrome (SIRS) not sustained by an infectious cause, particularly in the first days after trauma. Burns involving > 20% of TBSA are associated with massive release of inflammatory mediators and catecholamines, resulting in a hypermetabolic response, vasodilation, increased vascular permeability, fluid loss, hyperpyrexia, tachycardia, tachypnea, and hyperleukocytosis.

This condition can eventually lead to shock [31,32]. In this context, it can be challenging to diagnose septic complications, particularly in the early post-burn phase, when the vast majority of patients (with and without infectious complications) fulfill the criteria for SIRS [33]. Therefore, the use of SIRS criteria is formally discouraged by the Surviving Sepsis After Burn Campaign for identifying patients with sepsis [34]. The overarching definition of sepsis and septic shock, as proposed by The Third International Consensus Definitions for Sepsis and Septic Shock (Sepsis-3) [35], is also applicable to burn patients [34], but the main clinical challenge remains the early identification of sepsis in the burn setting. Different criteria have been proposed to help clinicians in this regard, but their accuracy is not optimal, and they require further validation (Table 2) [36,37,38,39].

A cautious use and interpretation of biomarkers, particularly procalcitonin, could provide additional guidance in discriminating burn patients with septic complications [38,40,41]. In a systematic review and meta-analysis of 28 studies, procalcitonin showed a sensitivity of 73% and a specificity of 75%. As shown in Table 2, according to the Surviving Sepsis After Burn Campaign, a procalcitonin increase of ≥2 ng/mL from the initial level can be considered as a signal of ongoing sepsis [34]. In contrast, C-reactive protein and hyperleukocytosis showed very low specificity (less than 50%) [42].

Bloodstream infections more frequently derive from cutaneous sources (wounds and vascular catheters) [43,44]. Several studies have shown that BSIs are often associated with poor prognosis, and the mortality rate is up to four times higher than in burn patients who do not develop bacteremia. Bloodstream infections are also associated with increased hospital length of stay, the need for vasopressors, and MV [44,45,46]. Pathogens isolated from wounds correlate only partially with those found in blood culture. Consequently, data on wound isolates can be considered when choosing empiric treatment for BSI, but cannot be the only element to inform this decision [47,48]. This can be particularly challenging when MDR pathogens are documented on wounds, since the decision to consider or disregard these pathogens can eventually lead to overtreatment or, conversely, to inappropriate antimicrobial coverage. In this setting, indications from other populations of critically ill patients may be extrapolated, including coverage for MDR pathogens in empiric treatment of severely ill, high-risk burn patients, with a systematic early de-escalation approach guided by aggressive microbiological sampling [49], as detailed in later paragraphs. In this context, the use of rapid molecular-based diagnostic tests performed on blood cultures may have a role [50].

Systemic antibiotic prophylaxis to prevent BSIs, as well as other systemic HAIs, is not recommended and should be avoided to reduce the risk of the emergence of MDR pathogens [36,51] (see later paragraphs for details).

### 4.2. Pneumonia

Pneumonia (including ventilator-associated pneumonia, VAP) is probably the most prevalent HAI in patients hospitalized for burn injury. This complication is particularly frequent and severe in patients with associated inhalation injury and prolonged MV [36,52]. The 2016 report of the NBR described an overall prevalence of 5.4% [26].

Overall, the management of VAP in burn patients should follow the best practices established for other critically ill patients [36]. Concerning microbiological diagnosis, obtaining appropriate samples through subglottic specimens, preferentially bronchoalveolar lavage (BAL), is suggested [36,53]. There is a poor association between wound colonizers and pathogens found in respiratory specimens; thus, the knowledge of skin colonizers is generally non-informative for guiding antibiotic treatment for VAP [54].

### 4.3. Wound Infections

**Figure 1:**
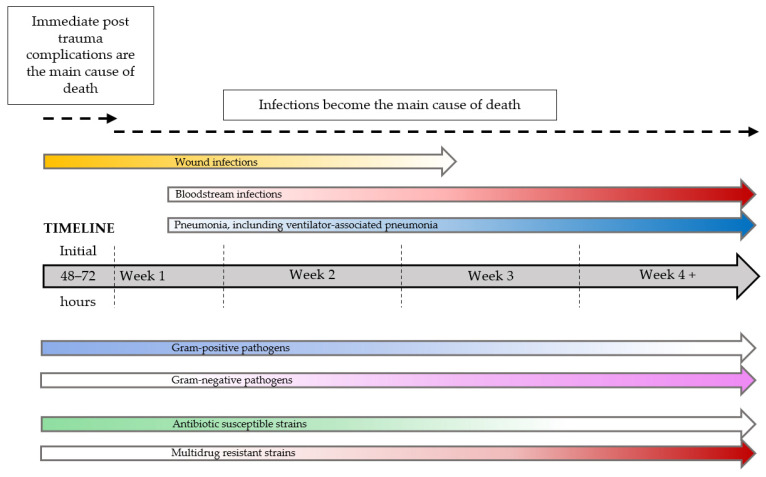
Infection complications according to the timeline after burn injury.

The loss of the skin barrier, together with the presence of damaged, inflamed, and partially necrotic tissue, makes the wound colonization unavoidable [55]. The wound can later develop a clinically relevant infection, which in turn compromises wound healing and sometimes leads to systemic infection [56,57]. Wound infections are often the first infectious complications encountered after hospital admission for burn injury, while pneumonia and BSIs tend to be more delayed [16,30] (Figure 1).

Infected wounds need to be managed aggressively. Early and extensive debridement of necrotic areas and appropriate dressing are of paramount importance [56,58]. Microbiological documentation with wound culture should always be attempted, but only deep, surgically obtained specimens should be analyzed to identify true pathogens and avoid skin colonizers that can lead to inappropriate antibiotic treatments. Unlike other settings, such as skin and soft tissue infections in non-burn patients and pressure ulcer infections, current guidelines encourage the use of topical antimicrobial agents (such as silver-based topical agents, e.g., silver sulfadiazine) in infected wounds in burn patients, with careful consideration of the risk of impaired wound healing [36,59].

## 5. Microbiology of Bacterial Hospital-Acquired Infections

The knowledge of epidemiology in each setting is an essential element for informing empiric antibiotic treatment in the case of HAIs.

The epidemiology of bacterial isolates and their resistance patterns is a dynamic phenomenon, changing significantly with the progression of the hospital stay [19,30] and the related use of invasive devices and antibiotic treatments [6,60] (Figure 1). During the first week after burn injury, wound infections predominate, and HAIs are mainly sustained by Gram-positive pathogens, particularly *Staphylococcus aureus*, and to a lesser extent other coagulase-negative Staphylococci (CoNS), Streptococci, and Enterococci [61]. After 5 to 7 days, there is a progressive increase in the prevalence of pneumonia (including VAP) and BSIs. In parallel, Gram-negative pathogens become more prevalent and finally predominant, particularly after the third week of hospitalization [30]. These bacteria derive from the host’s normal flora and/or from the hospital environment. The most represented are Enterobacterales, *Pseudomonas aeruginosa*, *Acinetobacter baumannii*, and *Stenotrophomonas maltophilia* [6,19,43,45,46,62,63]. In a large retrospective study by van Duin et al., reporting the epidemiology of HAIs in 5524 patients, with 1788 bacterial isolates, the median time from hospital admission to first positive culture was 2 days for Streptococci, 3 days for *Staphylococcus aureus* and 6.5 days for other Gram-positive pathogens, versus 11.5 days for Enterobacterales, 18 days for *Pseudomonas aeruginosa* and 26 days for *Acinetobacter baumannii* [30]. In case of prolonged hospitalization, fungal infections are also encountered more frequently (*Candida* spp., *Aspergillus* spp., *Fusarium* spp., *Mucorales*), particularly in the presence of multiple risk factors [63,64,65].

The distribution of MDR pathogens follows a similar dynamic, being influenced by the length of hospital stay (Figure 1). In the aforementioned large study by van Duin et al., 44% of isolated strains were MDR, and the median time from hospital admission to first positive culture was 11 days for non-MDR pathogens, versus 37 days for MDR pathogens. This trend was consistent for methicillin-resistant *Staphylococcus aureus* (MRSA); fluoroquinolone-resistant, extended-spectrum beta-lactamase (ESBL)-producing and carbapenem-resistant Enterobacterales (CRE); MDR *Pseudomonas aeruginosa*; and vancomycin-resistant *Enterococcus faecium* (VRE) [30]. Similarly, *Acinetobacter baumannii* and *Stenotrophomonas maltophilia*, which display MDR phenotypes predominantly, emerged after several weeks of hospitalization [30]. More recent findings, such as those reported by Cleland et al., showed a shorter median time from hospital admission to first positive MDR culture (11 days) and confirmed that Gram-negative MDR pathogens tend to emerge later, during hospital stay [10]. Despite the variation in MDR prevalence in other studies, according to the epidemiological setting, this association between the length of hospitalization and the emergence of MDR has been consistently confirmed in many other contexts [6,18,20,23,45,66,67,68,69,70,71].

Apart from these stable trends associated with the length of hospitalization, MDR pathogens can also spread in the form of outbreaks [72,73]. Outbreaks have been described in numerous settings and for all the most relevant pathogens, including MRSA [61,74], VRE [75], carbapenem-resistant Enterobacterales [76], MDR *Pseudomonas aeruginosa* [77,78], and carbapenem-resistant *Acinetobacter baumannii* [79].

Table 3 shows the main microbiological characteristics of the principal pathogens associated with HAIs in burn patients.

## 6. Antibiotic Treatment of Hospital-Acquired Infections in Burn Patients

### 6.1. General Considerations, Empiric Regimens and Targeted Therapies

**Figure 2:**
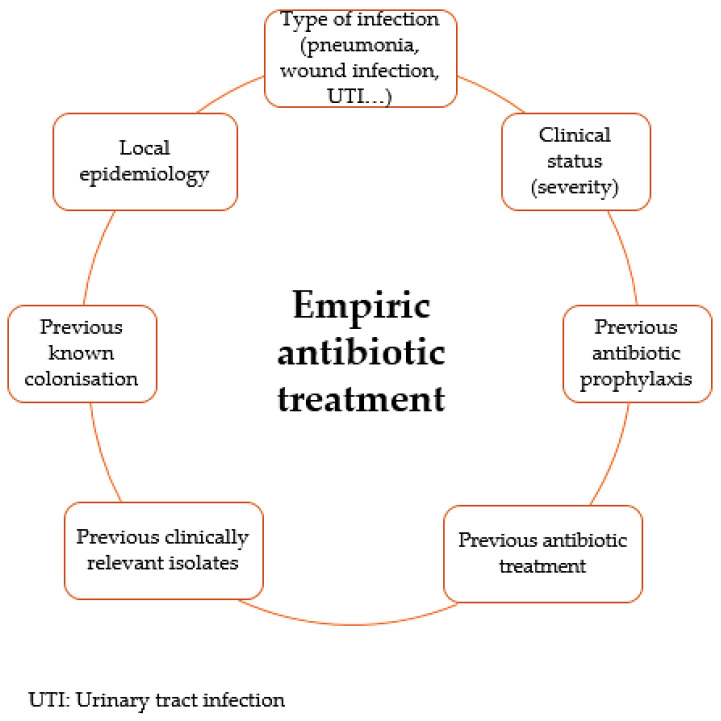
Factors influencing empiric antibiotic treatment.

When HAIs are suspected in burn patients, they require prompt and aggressive diagnostic and therapeutic management [36]. Hospital-acquired infections are associated with substantial morbidity and mortality, and empiric treatment is needed in most cases to avoid worse outcomes due to delays in active treatment [80,81]. As in other critical care settings, broad-spectrum antibiotics are needed to treat HAIs in burn patients, since MDR bacteria are highly prevalent (particularly Gram-negative pathogens), as discussed above. Many factors need to be taken into account when prescribing empiric antibiotic treatment, such as clinical severity, site of infection, local epidemiology, history of patient colonization, and previous antibiotic prophylaxis and/or treatment (Figure 2) [82,83,84].

The evaluation of clinical stability is the first step, since it determines how aggressive empiric antibiotic treatment should be. In more severe patients, such as those with sepsis and septic shock, a delay in initiating active antibiotic treatment is associated with increased mortality [85,86]. However, in burn patients, the definition of sepsis can be challenging, as discussed in paragraph 4.1 [37]. Several severity scores have been validated in patients with suspected or proven infection (such as the Sequential Organ Failure Assessment score (SOFA), the quick Sequential Organ Failure Assessment score (qSOFA), the National Early Warning Score (NEWS), and the National Early Warning Score 2 (NEWS2) [87,88,89]. These scores are intended to assess severity and risk of death at different time points and in different healthcare settings, helping clinicians establish how aggressive clinical and therapeutic management should be. However, little data are available in the setting of severely burned patients, and these scores should be interpreted with caution in this population. The Baux score and revised Baux score have been proposed to stratify the overall risk of death in burn patients, but they are also useful in the case of HAIs [17,90,91,92,93,94,95,96].

Recent exposure to antibiotic prophylaxis and/or antibiotic treatment should also be taken into account when choosing empiric antibiotic therapy, since it is a risk factor for infections sustained by MDR pathogens [21,22], as in other critical care settings [24,25]. Moreover, colonization by MDR pathogens is associated with an increased risk of developing a systemic infection (particularly bacteremia) sustained by those difficult-to-treat pathogens. Colonization should be screened with rectal, cutaneous, or nasal swabs, depending on the pathogen of interest. The negative predictive value of these tests is very high, while their positive predictive value varies according to the pathogen detected and the clinical context. Overall, in different studies, the percentage of colonized patients further developing a BSI sustained by the colonizing pathogen ranged from approximately 2 to 85%, depending on the setting and concomitant risk factors [97,98,99,100,101]. In severely burned patients, there is a well-demonstrated association between skin colonization by MDR pathogens and the risk of systemic infections caused by the same microorganisms [67]. Also, rectal carriage of MDR Enterobacterales and nasal carriage of MRSA are associated with the risk of MDR infections [47,48,102,103].

According to clinical severity at presentation (and considering the history of previous antibiotic treatment and known colonization), the treating physician can decide to follow a de-escalation or an escalation approach. A de-escalation approach is advisable in all cases with severe clinical presentation, e.g., sepsis or septic shock, and also in patients presenting with relevant clinical instability because of other concomitant conditions, such as patients with extensive burn [82]. It consists of starting with a broad-spectrum coverage, often including a combination of antibiotics targeting both resistant Gram-negative and Gram-positive pathogens (+/− an antifungal agent), which also considers known colonization, such as rectal colonization with CRE [82]. Then, when relevant microbiological results become available, the treatment is de-escalated, switching to a narrower-spectrum antibiotic and discontinuing agents that are not needed [104,105,106]. Due to the usual clinical severity of burn patients, this approach is the most suitable in many cases [34,82]. The escalation approach can be considered in patients who are judged clinically stable, particularly those with localized wound infections: a relatively narrower-spectrum antibiotic is prescribed as first-line therapy, and the treatment is promptly escalated to a broader-spectrum regimen (sometimes with a combination of two agents) in case of clinical deterioration or relevant microbiological findings [107]. These approaches have been extensively explored in the general population and are suggested by international guidelines, while they have been poorly explored in the burn setting.

**Figure 3:**
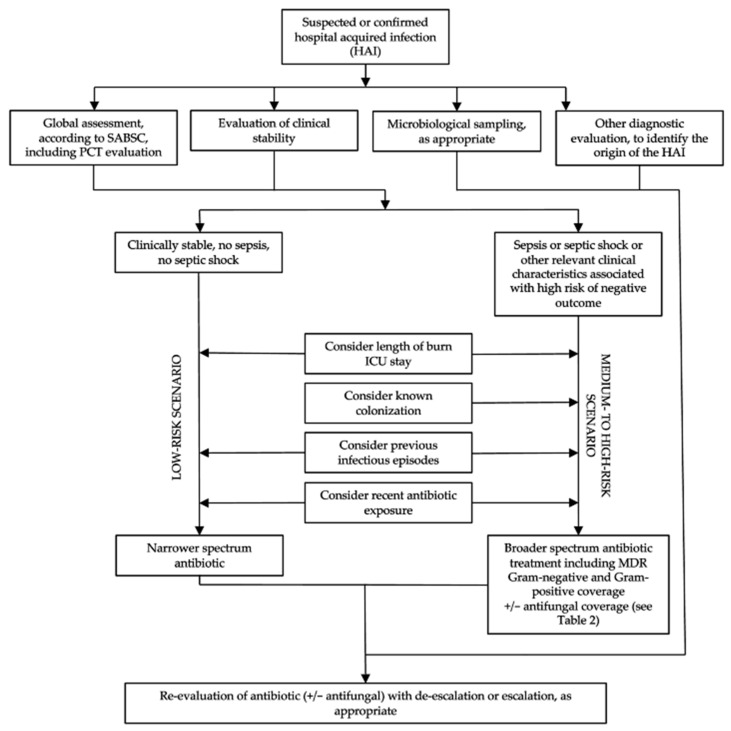
Proposed algorithm to guide initial empiric antimicrobial treatment in case of suspected/confirmed HAI in burn patients. PCT: procalcitonin; SSABC: Surviving Sepsis After Burn Campaign.

Figure 3 and Table 4 present an algorithm to guide empiric antibiotic treatment in burn patients with suspected or confirmed HAI. Table 5 lists first-line agents for targeted therapy in the case of the most commonly encountered isolates.

### 6.2. Pharmacokinetic/Pharmacodynamics Issues

Severe burns cause a significant alteration of normal homeostasis, due to the dramatic and dynamic pathophysiological events triggered by tissue disruption and consequent hyper-inflammatory response. These changes can include altered fluid balance with hemodynamic instability and rapid changes in volume of distribution, protein loss, and augmented renal clearance (ARC). Inter-patient variability can be substantial, depending on burn severity and host response, and even intra-patient variability is difficult to predict, since all these phenomena evolve rapidly, particularly during the first days after burn [111,112,113,114].

In the last few years, the understanding of optimal pharmacokinetics/pharmacodynamics (PK/PD) of antibiotics has significantly evolved, and new, more aggressive PK/PD targets have been proposed to achieve the highest probability of clinical cure and to reduce the risk of selection of resistant strains, particularly in critically ill patients [115,116,117]. For beta-lactams, which are the cornerstone of antibiotic treatment for septic burn patients, the suggested PK/PD target for severe infections is at least 100% *f*T/MIC (i.e., a free-drug plasma concentration higher than the minimum inhibitory concentration, for 100% of the time). Recent evidence suggests that a more ambitious target, such as 100% *f*T/4 × MIC (i.e., a free-drug concentration higher than 4-fold the minimum inhibitory concentration, for 100% of the time), may be associated with better outcomes [115,116].

In a recent systematic review of pharmacological evidence, our study group identified 35 studies assessing the pharmacological properties of broad-spectrum beta-lactam antibiotics in adult burn patients [9]. We showed that usual antibiotic dosages and administration modalities may be inadequate to achieve aggressive PK/PD targets in burn patients, particularly for severe infections such as pneumonia or BSIs.

In this context, higher doses and optimized administration modalities (i.e., prolonged or continuous infusion) are often needed to optimize antibiotic treatment [9,17,118,119,120,121]. Table 6 shows the suggested doses and administration modalities of 13 broad-spectrum beta-lactams, according to this evidence from the literature. Very few data are available on new beta-lactam-beta-lactamase inhibitor combinations, ceftaroline, ceftobiprole, and cefiderocol [122], as well as on antibiotic penetration in injured tissues [123].

Therapeutic drug monitoring (TDM) can be a useful tool in this setting to both optimize antibiotic exposure and reduce antibiotic-related toxicities [9,115]. Some experiences have been reported regarding the implementation of TDM in the setting of burn patients (particularly for beta-lactams [124,125] and vancomycin [121,126]), with promising results.

## 7. Antimicrobial Stewardship Considerations in HAIs in Burn Patients

As already underlined in the previous paragraphs, Burn ICUs are characterized by a high prevalence of MDR pathogens. This is closely interrelated to a high antibiotic consumption, particularly of broad-spectrum antibiotics, which needs to be carefully managed [127,128]. Antimicrobial stewardship (AMS) is an organized set of actions aimed at promoting appropriate and responsible antimicrobial use, with the objective of optimizing care for patients, avoiding at the same time the negative consequences of antibiotic overuse and misuse at the patient- and community-level [129]. Intensive care units are a challenging setting in which to implement AMS principles, due to the severity and rapidly evolving clinical presentation and the challenging epidemiology [130]. Nevertheless, many AMS interventions have been implemented in the ICU setting in the last two decades, showing the ability to reduce antibiotic consumption (both in absolute terms and in terms of broad-spectrum agents), improve the appropriateness of prescriptions, reduce the prevalence of MDR-associated infections, and reduce costs [121,122,123,124,125,126,127,128]. The most relevant AMS interventions applicable in the ICU setting are summarized in Table 7.

No specific guidance for antimicrobial stewardship in Burn Units exists. However, the ISBI Practice Guidelines for Burn Care suggest that an AMS program should be implemented in Burn Units [51]. These guidelines also provide several indications aimed at optimizing antibiotic use in this setting. Below is a quick summary of these indications [51], with some considerations regarding supporting evidence:

- Prophylactic systemic antibiotics should not be prescribed in burn patients. This is further detailed as follows:

> in the management of burn wounds, as they do not prevent burn colonization or infection [141,142,143]. To the contrary, topical antimicrobial agents are indicated in most cases [36].

> in patients considered at high risk for pneumonia, including those with inhalation injury, and those who are intubated [141,142,143,144].

- Microbiological specimens should always be sent for culture when starting an empiric antibiotic treatment to allow further tailoring of therapy. This includes subglottic respiratory specimens (preferably BAL) for respiratory infections [36,145].

- Once culture results are available, antibiotic de-escalation is encouraged. As already noted, this strategy has proven to be safe and effective in reducing antibiotic exposure, MDR carriage, and/or infections and costs, also in high-risk settings, such as neutropenic patients and ICU patients [104,105,107]. It remains poorly explored in the population of burn patients [106].

- Antibiotic duration should follow recommendations for other critically ill populations. In this regard, the ISBI Guidelines provide details only on VAP [36]. Antibiotic treatment of 7 days for bacteremia and 7 days or less for pneumonia is strongly supported by evidence in the general population [146,147,148,149]. Although there is a paucity of data in burn patients, the quality of this RCT-based evidence suggests that this approach should be safely applied in this setting.

Apart from these clinical indications (which aim to promote judicious antibiotic use in Burn Units), few data are available on specific integrated AMS interventions in the setting of burn patients. These are mainly before–after studies, often with a moderate-to-high risk of bias, mainly due to their observational design and, in some cases, to the small population size. Nauriyal et al. [150] performed a before–after study focusing on the implementation of a post-prescription review and feedback program, involving a total of 477 burn patients. They showed an improvement in indicators of judicious antibiotic use, such as de-escalation, accurate documentation in the patient’s chart, and adherence to antibiotic prescribing guidelines. However, the reduction in antibiotic consumption concerned only certain classes, and an increase in fluoroquinolones use was noted. Finally, outcome indicators (e.g., mortality) were not reported.

Fonseca-Rivera et al. [151] performed a before–after analysis exploring the effect of an AMS intervention based on formulary restrictions and systematic AMS specialist advice on antibiotic prescribing in a population of children hospitalized in different ICUs. They noted a reduction in antibiotic consumption, with no detrimental effect on mortality. Of note, the population of burn children was substantial (260 patients in total) and corresponded to the subgroup characterized by the highest baseline antibiotic consumption. Sadeq et al. [152] showed a reduction in length of hospital stay, readmission, consumption of reserve antibiotics, and mortality after the implementation of an AMS intervention based on an AMS multidisciplinary team. However, the population of this study was heterogeneous, including non-critically and critically ill patients, with only 25 burn patients in the intervention group. Zbyrak et al. [153] showed a benefit in terms of antibiotic consumption after the implementation of a PCT-guided algorithm in a Burn Unit, but the sample size was very small.

In conclusion, AMS programs are undoubtedly critical in Burn Units, where high antibiotic consumption, high prevalence of MDR-bacteria, and high overall and infection-related mortality occur. Nonetheless, currently there is very little evidence on AMS implementation and its impact, and further studies are urgently needed.

## 8. Conclusions

Hospital-acquired infections are a leading cause of morbidity and mortality in burn patients. Their management is challenging and requires a flexible approach, as diagnostic and therapeutic challenges evolve throughout prolonged hospitalization in Burn Units. A close multidisciplinary collaboration among burn experts (dermatologists, reconstructive surgeons, intensive care physicians), infectious disease specialists, microbiologists, and clinical pharmacologists is essential to achieve the best outcomes for patients. Further studies are needed on several aspects of the management of infected burn patients:

(i) Improving the accuracy of diagnostic criteria for suspected sepsis (particularly in the early stages after burn injury)
(ii) Better defining the role of colonizing pathogens in guiding antimicrobial empiric treatment for systemic infections
(iii) Expanding our understanding of PK/PD properties of antibiotics in burn patients, particularly for novel molecules used to treat MDR pathogens
(iv) Identifying the best way to implement effective and sustainable AMS programs

## Figures and Tables

**Table 1:** Principal search strings used in MEDLINE.

(“Burns”[Mesh]) AND “Infections”[Mesh] - Filter for publication date 2000–2025 - Filter for review - Filter for randomised controlled trial - Filter for systematic review - Filter for meta-analysis
(“Burns”[Mesh]) AND “Infections”[Mesh] and epidemiology
(“Burns”[Mesh]) AND (“Infections”[Mesh]) and (“multidrug resistant” OR “multi-drug resistant”) - Filter for publication date 2000–2025 - Filter for review - Filter for randomised controlled trial - Filter for systematic review - Filter for meta-analysis
“Burns” AND “Infections” and (“pharmacokinetic/pharmacodynamic”) OR (“pharmacokinetic/pharmacodynamics”)
(“Infections”[Mesh]) AND “Burns”[Mesh] AND “tissue penetration”
(“Burns”[Mesh]) AND (“Infections”[Mesh]) and (“antibiotic stewardship” OR “antimicrobial stewardship”)
“Burns” AND “Infections” and (“antibiotic stewardship” OR “antimicrobial stewardship”)
(“Infections”[Mesh]) AND “Burns”[Mesh] AND guidelines
qSOFA AND Burn
SOFA AND Burn
(“Organ Dysfunction Scores”[Mesh]) AND “Burns”[Mesh]
NEWS AND Burn
NEWS2 AND Burn
“Baux score” and mortality
“Revised Baux score” AND mortality OR “rBaux score” AND mortality
(“Burns”[Mesh]) AND “Infections”[Mesh] AND “revised baux score”
(“Burns”[Mesh]) AND “Infections”[Mesh] AND (“colonization” OR “colonisation”)
(“burn”[tiab] or “burns”[tiab] or “burned”[tiab]) and (“pharmacokinetic” AND/OR “pharmacodynamics”)
(“antibiotic stewardship” or “antimicrobial stewardship”) AND (“burn”[tiab] or “burns”[tiab])

**Table 2:** Proposed criteria for the definition of sepsis in adult burn patients ^¥^.

Society or First Author	ABA [37], Endorsed by ISBI [36]	Greenhalgh DG et al. [34], Surviving Sepsis After Burn Campaign, Endorsed by ISBI
Year of publication	ABA: 2007 ISBI: 2017	2023
Criteria	Temperature: >39 °C or <36.5 °C Progressive tachycardia: >110 beats/min Progressive tachypnea > 25 breaths/min or minute ventilation > 12 L/min Thrombocytopenia < 100,000/mcL (does not apply until 3 days after burn) Hyperglycemia in the absence of pre-existing diabetes mellitus (Untreated plasma glucose > 200 mg/dL or intravenous insulin > 7 units/h IV, significant resistance to insulin [>25% increase in insulin requirements over 24 h]) Inability to continue enteral feedings > 24 h (Abdominal distension, enteral feeding intolerance [two times feeding rate], uncontrollable diarrhea [>2500 mL/day])	Change in SOFA ≥ 2 points Lactate change > 2 mmol/L (>18 mg/dL) Temperature change: new fever or hypothermia (no consensus on threshold temperature) Acute drop in platelet count Urine output drop/increased fluid requirements Kidney Disease Improving Global Outcomes (KDIGO) Acute Kidney Injury Stage ≥ 1 Respiratory changes Alterations of mental status Gastrointestinal dysfunction Change in wound appearance suggestive of infection Procalcitonin increase ≥ 2 ng/mL from initial level
Interpretation	Diagnosis of sepsis requires ≥3 criteria plus at least one of the following features of infection: Positive culture Pathologic tissue source Clinical response to antimicrobials	These findings should be considered triggers for considering a diagnosis of sepsis in burn patients. No single criterion is sufficient to establish the diagnosis; rather, the presence of multiple criteria more reliably predicts sepsis and indicates the need for further diagnostic evaluation and therapeutic intervention.

¥: Adapted from ISBI Practice Guidelines for Burn Care [36] and Surviving Sepsis After Burn Campaign [34]; ABA: American Burn Association; ISBI: International Society for Burn Injuries.

**Table 3:** Principal pathogens implicated in hospital-acquired infections in burn patients.

Pathogens	Microbiological Characteristics	Main Infectious Syndromes in Burn Patients *	Main Acquired Phenotypic Resistance Profile
*Staphylococcus aureus*	Facultatively anaerobic, Gram-positive cocci, typically arranged in clusters	Bacteremia, including CVC infections Wound infections Pneumonia	Methicillin-resistance, determining resistance to all beta-lactams (except ceftaroline and ceftobiprole); resistance to fluoroquinolones
Coagulase-negative Staphylococci (CoNS)	Facultatively anaerobic, Gram-positive cocci, typically arranged in clusters	Bacteremia, including CVC infections Wound infections Much less virulent than *S. aureus*	Methicillin-resistance, determining resistance to all beta-lactams (except ceftaroline and ceftobiprole); resistance to fluoroquinolones
Streptococci	Facultatively anaerobic, Gram-positive cocci, typically arranged in chains or pairs	Bacteremia, including CVC infections, Wound infections Pneumonia Intra-abdominal infections	Resistance to ampicillin (in some species); resistance to 3GC is described, but overall rare (and only in some species) Resistance to macrolides and lincosamides
*Enterococcus faecalis*	Facultatively anaerobic, Gram-positive cocci, typically arranged in short chains or pairs	Bacteremia, including CVC infections IAIs UTIs	Lack of synergy between beta-lactams and aminoglycosides
*Enterococcus faecium*	Facultatively anaerobic, Gram-positive cocci, typically arranged in short chains or pairs	Bacteremia, including CVC infections IAIs UTIs	Resistance to ampicillin Resistance to vancomycin
Enterobacterales (order of bacteria, including *Escherichia coli*, *Klebsiella pneumoniae*, *Enterobacter cloacae*, *Serratia marcescens*, *Morganella morganii*, *Salmonella* spp., and many others)	Facultatively anaerobic, Gram-negative bacilli	Bacteremia, including CVC infections Pneumonia Wound infections IAIs UTIs	Resistance to 3GC Resistance to carbapenems
*Pseudomonas aeruginosa*	Aerobic, Gram-negative bacilli	Bacteremia, including CVC infections Pneumonia Wound infections IAIs UTIs	Complex combination of resistance mechanisms, determining multiple resistance patterns, including resistance to ceftazidime and cefepime, resistance to piperacillin–tazobactam, resistance to carbapenems, resistance to new beta-lactam-beta-lactamase inhibitor combinations, and cefiderocol
*Acinetobacter baumannii*	Aerobic, Gram-negative short bacilli, sometimes appearing as coccobacilli	Bacteremia, including CVC infections Pneumonia Wound infections IAIs UTIs	Carbapenem resistance

3GC: third generation cephalosporins; CVC: central venous catheter; IAIs: intra-abdominal infections; UTIs: urinary tract infection. * Sepsis and septic shock can derive from each of these infectious syndromes, more commonly when the infection is sustained by more virulent pathogens, e.g., *Staphylococcus aureus*, some Streptococci, Enterobacterales, *Pseudomonas aeruginosa*.

**Table 4:** Suggested initial empiric antimicrobial treatment in case of suspected/confirmed HAI in burn patients.

Clinical Scenario	Other Clinically Relevant Features	Empiric Antibiotic Treatment	Notes
Low-risk scenario (see Figure 3)	First infectious episode, first 2 weeks of hospitalization	TZP + daptomycin or vancomycin	In context with the high prevalence of 3GC resistance, consider meropenem instead of TZP, particularly if TZP resistance is frequent as well.
	First infectious episode, ≥2 weeks of hospitalization	TZP	
	In case of pneumonia (including VAP)	Add anti-Gram-positive coverage (linezolid or vancomycin) irrespective of length of hospitalization	Avoid daptomycin, since it is inactivated by the surfactant [108]. Perform a nasal swab for MRSA if this is not already available. If the nasal swab for MRSA is negative, discontinue anti-Gram-positive coverage
	In case of wound infection	Add anti-Gram-positive coverage irrespective of the length of hospitalization	
	In case of UTI	Avoid anti-Gram-positive coverage (daptomycin or vancomycin) irrespective of the length of hospitalization	
Medium- to high-risk scenario (see Figure 3)	First infectious episode, first 2 weeks of hospitalization	Meropenem + daptomycin or vancomycin	
	First infectious episode, ≥2 weeks of hospitalization	Meropenem + daptomycin or vancomycin + an echinocandin	
	Known KPC-producing Enterobacterales colonization	Ceftazidime–avibactam or meropenem–vaborbactam or imipenem–cilastatin–relebactam + daptomycin or vancomycin + an echinocandin	
	Known MBL-producing Enterobacterales colonization	Aztreonam–avibactam or ceftazidime–avibactam + aztreonam + daptomycin or vancomycin + an echinocandin	
	Known OXA-48-producing Enterobacterales colonization	Ceftazidime–avibactam + daptomycin or vancomycin + an echinocandin	
	Known MDR *Pseudomonas aeruginosa* colonization	Ceftolozane–tazobactam (if active) + daptomycin or vancomycin + an echinocandin	If there are other concomitant colonizations, they have to be taken into account (e.g., ceftolozane–tazobactam is inactive on carbapenemase-producing strains)
	In case of pneumonia (including VAP)	As before, but avoid daptomycin, preferring linezolid or vancomycin. Antifungal coverage (echinocandin) is not needed.	Avoid daptomycin, since it is inactivated by the surfactant. Perform a nasal swab for MRSA if this is not already available. If the nasal swab for MRSA is negative, discontinue anti-Gram-positive coverage
	In case of UTI	Avoid anti-Gram-positive coverage irrespective of the length of hospitalization	

3GC: third-generation cephalosporins. KPC: Klebsiella pneumoniae carbapenemase. MBL: metallo beta-lactamase. MRSA: methicillin-resistant Staphylococcus aureus. TZP: piperacillin–tazobactam. UTI: urinary tract infection. VAP: ventilator-associated pneumonia.

**Table 5:** Suggested targeted antimicrobial treatment in case of suspected/confirmed HAI [83,84,109,110].

**Gram-Positive Pathogens**	
Methicillin-susceptible *Staphylococcus aureus*	Cefazolin or (flu)(cl)oxacillin
Methicillin-resistant *Staphylococcus aureus*	Daptomycin, vancomycin, ceftaroline, or ceftobiprole For pneumonia: linezolid, vancomycin, ceftaroline, or ceftobiprole. Consider dalbavancin, particularly for wound infection and bone and joint infections
*Streptococcus pneumoniae*	Ampicillin or ceftriaxone/cefotaxime (according to AST)
Other Streptococci	Ampicillin, ceftriaxone, or vancomycin (according to AST)
*Enterococcus faecalis*	Ampicillin (+ceftriaxone for endocarditis)
*Enterococcus faecium*	Vancomycin
Vancomycin-resistant *Enterococcus faecium*	Linezolid or daptomycin
**Gram-negative pathogens**	
3GC-susceptible Enterobacterales	Ceftriaxone or cefotaxime
3GC-resistant Enterobacterales, ESBL producers	Stable patients and MIC for piperacillin–tazobactam ≤ 4 mg/L: piperacillin–tazobactam Unstable patients or MIC for piperacillin–tazobactam > 4 mg/L: meropenem
3GC-resistant Enterobacterales, inducible chromosomal AmpC-producers *	If MIC for cefepime ≤ 1 mg/L: cefepime If MIC for cefepime > 1 mg/L or ESBL co-production: meropenem
KPC-producing Enterobacterales	Ceftazidime–avibactam or meropenem–vaborbactam or imipenem–cilastatin–relebactam
MBL-producing Enterobacterales	Aztreonam–avibactam or ceftazidime–avibactam + aztreonam
OXA-48-producing Enterobacterales	Ceftazidime–avibactam
MDR *Pseudomonas aeruginosa*	Ceftolozane–tazobactam if active; otherwise, choose one fully active beta-lactam agent If no fully active beta-lactam (i.e., MIC at breakpoint) is present, consider the addition of a second non-beta-lactam agent If resistant to all beta-lactams, consider the association of two non-beta-lactam agents
Carbapenem-resistant *Acinetobacter baumannii*	Sulbactam–durlobactam + imipenem–cilastatin If sulbactam–durlobactam is not available: high dose ampicillin–sulbactam (≥9 g of sulbactam/day) + a second agent among colistin, cefiderocol, tigecycline, eravacycine, minocycline, fosfomycin (according to susceptibility test)
*Stenotrophomonas maltophilia*	Trimethoprim/sulfamethoxazole + levofloxacin. If one of these two agents is inactive, add a second agent among tigecycline, eravacycine, minocycline, cefiderocol (according to susceptibility test)
**Fungi**	
*Candida* spp.	Caspofungin, anidulafungin, or micafungin. Descalation to fluconazole is indicated once the fungemia is cleared and the patient is clinically stable, in susceptible strains. Consider rezafungin, particularly for long-course treatment.
*Candida auris*	Variable susceptibility profile, treat with caspofungin, anidulafungin, micafungin, or liposomal amphotericin B, according to susceptibility test. Seek urgent infectious diseases advice.
*Filamentous fungi*	Liposomal amphotericin B, voriconazole, or isavuconazole, according to isolated species and susceptibility tests. Seek urgent infectious diseases advice.

3GC: third-generation cephalosporins. AST: antibiotic susceptibility testing. ESBL: extended-spectrum beta-lactamases. MIC: minimum inhibitory concentration. *: *Enterobacter cloacae*, *Klebsiella aerogenes*, *Citrobacter freundii*.

**Table 6:** Suggested doses and administration modes for broad-spectrum beta-lactams in burn patients with severe burns.

Antibiotic	Suggested Drug Regimens in Burn Patients with Severe Infection
Ceftazidime	1 g every 4 h or CI of 6 g/24 h (with loading dose)
Cefepime	2 g every 8 h; consider CI of 6 g/24 h (with loading dose)
piperacillin–tazobactam	18 g/24 h CI (with loading dose). Higher doses may be needed for patients with ARC.
Meropenem	6 g/24 h CI (with loading dose). Higher doses may be needed for patients with ARC.
imipenem–cilastatin	500 mg every 6 h; 1 g every 6 h if ARC.
Aztreonam	2 g every 6–8 h or 6–8 g in CI (particularly if ARC)
ceftaroline ceftobiprole ceftolozane–tazobactam cefepime–enmetazobactam ceftazidime–avibactam meropenem–vaborbactam imipenem-cil.–relebactam aztreonam–avibactam cefiderocol	Insufficient data on burn patients. Standard regimens: - ceftaroline: 0.6 g every 8 h - ceftobiprile: 0.5 g every 8 h - ceftolozane–tazobactam: 3 g every 8 h - cefepime–enmetazobactam: 2.5 g every 8 h - ceftazidime–avibactam: 2.5 g every 8 h - meropenem–vaborbactam: 4 g every 8 h - imipenem–cilastatin–relebactam: 1.25 g every 6 h - aztreonam–avibactam: 2 g every 6 h - cefiderocol: 2 g every 8 h

Doses are intended for patients without renal impairment. In case of continuous infusion, the stability of the molecule needs to be taken into account (e.g., 6 g/24 h continuous infusion of meropenem = 2 g in 8 h 3 times a day). ARC: augmented renal clearance, i.e., estimated glomerular filtration rate > 120 mL/m. CI: continuous infusion. Adapted from Tebano G.et al. [9].

**Table 7:** Main antimicrobial stewardship intervention in ICU [131,132,133,134,135,136,137,138,139,140].

Institution of the AMS team
Education for prescribers
Preauthorization for reserve antibiotics and formulary restrictions
Systematic (unsolicited) audit and feedback on antibiotic use (review of antibiotic prescriptions and provision of feedback to prescribers)
Treatment guidance tailored to local epidemiology
Treatment algorithm tailored to patient colonization status (e.g., use of MRSA nasal swab to reduce anti-MRSA coverage, use CRE screening to guide anti-Gram-negative treatment)
Rapid molecular diagnostics (e.g., on blood cultures and respiratory samples)
Selective reporting of antibiotic susceptibility testing
Biomarker-guided antibiotic initiation or discontinuation protocols
Computerized clinical decision support tools (computer-assisted decision support)
Reassessment of antibiotic prescriptions on a pre-specified day of therapy (e.g., day 2, 3, 7)
Antibiotic de-escalation protocols
Algorithm guiding antibiotic use in end-of-life setting

AMS: antimicrobial stewardship. CRE: carbapenem-resistant Enterobacterales. MRSA: methicillin-resistant Staphylococcus aureus.

## Data Availability

No new data were created or analyzed in this study. Data sharing is not applicable to this article.

## Abbreviations

ABA	American Burn Association
AMS	Antimicrobial Stewardship
ARC	Augmented Renal Clearance
AST	Antibiotic Susceptibility Testing
BAL	Bronchoalveolar Lavage
BSI	Bloodstream Infection
CCI	Charlson Comorbidity Index
CI	Continuous Infusion
CRE	Carbapenem-Resistant Enterobacterales
CVC	Central Venous Catheter
CoNS	Coagulase-Negative Staphylococci
ESBL	Extended-Spectrum Beta-Lactamase
HAI	Hospital-Acquired Infection
ICU	Intensive Care Unit
ISBI	International Society for Burn Injuries
KPC	Klebsiella pneumoniae Carbapenemase
MBL	Metallo-Beta-Lactamase
MDR	Multidrug-Resistant
MIC	Minimum Inhibitory Concentration
MRSA	Methicillin-Resistant Staphylococcus aureus
MV	Mechanical Ventilation
NBR	National Burn Repository
NEWS	National Early Warning Score
NEWS2	National Early Warning Score 2
OXA	Oxacillinase
PK/PD	Pharmacokinetics/Pharmacodynamics
qSOFA	Quick Sequential Organ Failure Assessment
SIRS	Systemic Inflammatory Response Syndrome
SOFA	Sequential Organ Failure Assessment
TBSA	Total Body Surface Area
TZP	Piperacillin–Tazobactam
UTI	Urinary Tract Infection
VAP	Ventilator-Associated Pneumonia
VRE	Vancomycin-Resistant Enterococcus
WHO	World Health Organization

## References

[1] World Health Organization Burns. https://www.who.int/news-room/fact-sheets/detail/burns.

[2] Smolle C., Cambiaso-Daniel J., Forbes A.A., Wurzer P., Hundeshagen G., Branski L.K., Huss F., Kamolz L.-P. (2017). Recent Trends in Burn Epidemiology Worldwide: A Systematic Review. Burns.

[3] Guidelines for Burn Patient Referral—American Burn Association. https://ameriburn.org/resources/burnreferral/.

[4] Krishnan P., Frew Q., Green A., Martin R., Dziewulski P. (2013). Cause of Death and Correlation with Autopsy Findings in Burns Patients. Burns.

[5] Ressner R.A., Murray C.K., Griffith M.E., Rasnake M.S., Hospenthal D.R., Wolf S.E. (2008). Outcomes of Bacteremia in Burn Patients Involved in Combat Operations Overseas. J. Am. Coll. Surg..

[6] Lachiewicz A.M., Hauck C.G., Weber D.J., Cairns B.A., van Duin D. (2017). Bacterial Infections After Burn Injuries: Impact of Multidrug Resistance. Clin. Infect. Dis..

[7] Sun F.-J., Zhang X.-B., Fang Y., Chen J., Xing H., Shi H., Feng W., Xia P. (2012). Spectrum and Drug Resistance of Pathogens from Patients with Burns. Burns.

[8] Alp E., Coruh A., Gunay G.K., Yontar Y., Doganay M. (2012). Risk Factors for Nosocomial Infection and Mortality in Burn Patients: 10 Years of Experience at a University Hospital. J. Burn Care Res..

[9] Tebano G., la Martire G., Raumer L., Cricca M., Melandri D., Pea F., Cristini F. (2023). Which Are the Best Regimens of Broad-Spectrum Beta-Lactam Antibiotics in Burn Patients? A Systematic Review of Evidence from Pharmacology Studies. Antibiotics.

[10] Cleland H., Tracy L.M., Padiglione A., Stewardson A.J. (2022). Patterns of Multidrug Resistant Organism Acquisition in an Adult Specialist Burns Service: A Retrospective Review. Antimicrob. Resist. Infect. Control.

[11] van Langeveld I., Gagnon R.C., Conrad P.F., Gamelli R.L., Martin B., Choudhry M.A., Mosier M.J. (2017). Multiple-Drug Resistance in Burn Patients: A Retrospective Study on the Impact of Antibiotic Resistance on Survival and Length of Stay. J. Burn Care Res..

[12] Magiorakos A.-P., Srinivasan A., Carey R.B., Carmeli Y., Falagas M.E., Giske C.G., Harbarth S., Hindler J.F., Kahlmeter G., Olsson-Liljequist B. (2012). Multidrug-Resistant, Extensively Drug-Resistant and Pandrug-Resistant Bacteria: An International Expert Proposal for Interim Standard Definitions for Acquired Resistance. Clin. Microbiol. Infect..

[13] Dissanaike S., Ha D., Mitchell D., Larumbe E. (2017). Socioeconomic Status, Gender, and Burn Injury: A Retrospective Review. Am. J. Surg..

[14] Golshan A., Patel C., Hyder A.A. (2013). A Systematic Review of the Epidemiology of Unintentional Burn Injuries in South Asia. J. Public Health.

[15] Forjuoh S.N. (2006). Burns in Low- and Middle-Income Countries: A Review of Available Literature on Descriptive Epidemiology, Risk Factors, Treatment, and Prevention. Burns.

[16] Strassle P.D., Williams F.N., Weber D.J., Sickbert-Bennett E.E., Lachiewicz A.M., Napravnik S., Jones S.W., Cairns B.A., van Duin D. (2017). Risk Factors for Healthcare-Associated Infections in Adult Burn Patients. Infect. Control Hosp. Epidemiol..

[17] Corcione S., Pensa A., Castiglione A., Lupia T., Bortolaso B., Romeo M.R., Stella M., Rosa F.G.D. (2021). Epidemiology, Prevalence and Risk Factors for Infections in Burn Patients: Results from a Regional Burn Centre’s Analysis. J. Chemother..

[18] Escandón-Vargas K., Tangua A.R., Medina P., Zorrilla-Vaca A., Briceño E., Clavijo-Martínez T., Tróchez J.P. (2020). Healthcare-Associated Infections in Burn Patients: Timeline and Risk Factors. Burns.

[19] Wanis M., Walker S.A.N., Daneman N., Elligsen M., Palmay L., Simor A., Cartotto R. (2016). Impact of Hospital Length of Stay on the Distribution of Gram Negative Bacteria and Likelihood of Isolating a Resistant Organism in a Canadian Burn Center. Burns.

[20] Keen E.F., Robinson B.J., Hospenthal D.R., Aldous W.K., Wolf S.E., Chung K.K., Murray C.K. (2010). Prevalence of Multidrug-Resistant Organisms Recovered at a Military Burn Center. Burns.

[21] Cavalcante R.d.S., Canet P., Fortaleza C.M.C.B. (2014). Risk Factors for the Acquisition of Imipenem-Resistant Acinetobacter Baumannii in a Burn Unit: An Appraisal of the Effect of Colonization Pressure. Scand. J. Infect. Dis..

[22] Wibbenmeyer L., Williams I., Ward M., Xiao X., Light T., Latenser B., Lewis R., Kealey G.P., Herwaldt L. (2010). Risk Factors for Acquiring Vancomycin-Resistant Enterococcus and Methicillin-Resistant Staphylococcus Aureus on a Burn Surgery Step-down Unit. J. Burn Care Res..

[23] Javanmardi F., Emami A., Pirbonyeh N., Rajaee M., Hatam G., Keshavarzi A. (2019). Study of Multidrug Resistance in Prevalent Gram-Negative Bacteria in Burn Patients in Iran: A Systematic Review and Meta-Analysis. J. Glob. Antimicrob. Resist..

[24] Raman G., Avendano E.E., Chan J., Merchant S., Puzniak L. (2018). Risk Factors for Hospitalized Patients with Resistant or Multidrug-Resistant *Pseudomonas aeruginosa* Infections: A Systematic Review and Meta-Analysis. Antimicrob. Resist. Infect. Control.

[25] Diao H., Lu G., Zhang Y., Wang Z., Liu X., Ma Q., Yu H., Li Y. (2024). Risk Factors for Multidrug-Resistant and Extensively Drug-Resistant Acinetobacter Baumannii Infection of Patients Admitted in Intensive Care Unit: A Systematic Review and Meta-Analysis. J. Hosp. Infect..

[26] Kahn S.A., Bernal N., Mosier M.J. (2018). Pearls From the National Burn Repository. J. Burn Care Res..

[27] Tedesco D.J., Hutter M.F., Khalaf F., Ricciuti Z., Jeschke M.G. (2025). Sepsis in Burn Care: Incidence and Outcomes. Mil. Med. Res..

[28] Kelly E.J., Oliver M.A., Carney B.C., Shupp J.W. (2022). Infection and Burn Injury. Eur. Burn J..

[29] Norbury W., Herndon D.N., Tanksley J., Jeschke M.G., Finnerty C.C. (2016). Infection in Burns. Surg. Infect..

[30] van Duin D., Strassle P.D., DiBiase L.M., Lachiewicz A.M., Rutala W.A., Eitas T., Maile R., Kanamori H., Weber D.J., Cairns B.A. (2016). Timeline of Health Care-Associated Infections and Pathogens after Burn Injuries. Am. J. Infect. Control.

[31] Bacomo F.K., Chung K.K. (2011). A Primer on Burn Resuscitation. J. Emerg. Trauma Shock.

[32] Dg G. (2017). Sepsis in the Burn Patient: A Different Problem than Sepsis in the General Population. Burn. Trauma.

[33] Dastagir K., Krezdorn N., Bingoel A.S., Mett T.R., Radtke C., Jokuszies A., Vogt P.M. (2021). Application of Critical Care Scores in Severely Burned Patients. J. Burn Care Res..

[34] Greenhalgh D.G., Hill D.M., Burmeister D.M., Gus E.I., Cleland H., Padiglione A., Holden D., Huss F., Chew M.S., Kubasiak J.C. (2023). Surviving Sepsis After Burn Campaign. Burns.

[35] Singer M., Deutschman C.S., Seymour C.W., Shankar-Hari M., Annane D., Bauer M., Bellomo R., Bernard G.R., Chiche J.-D., Coopersmith C.M. (2016). The Third International Consensus Definitions for Sepsis and Septic Shock (Sepsis-3). JAMA.

[36] ISBI Practice Guidelines Committee, Advisory Subcommittee, Steering Subcommittee (2018). ISBI Practice Guidelines for Burn Care, Part 2. Burns.

[37] Greenhalgh D.G., Saffle J.R., Holmes J.H., Gamelli R.L., Palmieri T.L., Horton J.W., Tompkins R.G., Traber D.L., Mozingo D.W., Deitch E.A. (2007). American Burn Association Consensus Conference to Define Sepsis and Infection in Burns. J. Burn Care Res..

[38] Mann-Salinas E.A., Baun M.M., Meininger J.C., Murray C.K., Aden J.K., Wolf S.E., Wade C.E. (2013). Novel Predictors of Sepsis Outperform the American Burn Association Sepsis Criteria in the Burn Intensive Care Unit Patient. J. Burn Care Res..

[39] Stanojcic M., Vinaik R., Jeschke M.G. (2018). Status and Challenges of Predicting and Diagnosing Sepsis in Burn Patients. Surg. Infect..

[40] Cabral L., Afreixo V., Almeida L., Paiva J.A. (2016). The Use of Procalcitonin (PCT) for Diagnosis of Sepsis in Burn Patients: A Meta-Analysis. PLoS ONE.

[41] Cabral L., Afreixo V., Meireles R., Vaz M., Chaves C., Caetano M., Almeida L., Paiva J.A. (2018). Checking Procalcitonin Suitability for Prognosis and Antimicrobial Therapy Monitoring in Burn Patients. Burn. Trauma.

[42] Li A.T., Moussa A., Gus E., Paul E., Yii E., Romero L., Lin Z.C., Padiglione A., Lo C.H., Cleland H. (2022). Biomarkers for the Early Diagnosis of Sepsis in Burns: Systematic Review and Meta-Analysis. Ann. Surg..

[43] Patel B.M., Paratz J.D., Mallet A., Lipman J., Rudd M., Muller M.J., Paterson D.L., Roberts J.A. (2012). Characteristics of Bloodstream Infections in Burn Patients: An 11-Year Retrospective Study. Burns.

[44] Raz-Pasteur A., Hussein K., Finkelstein R., Ullmann Y., Egozi D. (2013). Blood Stream Infections (BSI) in Severe Burn Patients--Early and Late BSI: A 9-Year Study. Burns.

[45] Shupp J.W., Pavlovich A.R., Jeng J.C., Pezzullo J.C., Oetgen W.J., Jaskille A.D., Jordan M.H., Shoham S. (2010). Epidemiology of Bloodstream Infections in Burn-Injured Patients: A Review of the National Burn Repository. J. Burn Care Res..

[46] Fochtmann-Frana A., Freystätter C., Vorstandlechner V., Barth A., Bolliger M., Presterl E., Ihra G., Muschitz G., Mittlboeck M., Makristathis A. (2018). Incidence of Risk Factors for Bloodstream Infections in Patients with Major Burns Receiving Intensive Care: A Retrospective Single-Center Cohort Study. Burns.

[47] Gallaher J.R., Banda W., Lachiewicz A.M., Krysiak R., Cairns B.A., Charles A.G. (2018). Colonization with Multidrug-Resistant Enterobacteriaceae Is Associated with Increased Mortality Following Burn Injury in Sub-Saharan Africa. World J. Surg..

[48] Pangli H., Papp A. (2019). The Relation between Positive Screening Results and MRSA Infections in Burn Patients. Burns.

[49] Final Recommendations of the ECIL 10 Working Group. https://www.ecil-leukaemia.com/en/resources/resources-ecil.

[50] Dunbar S.A., Gardner C., Das S. (2022). Diagnosis and Management of Bloodstream Infections with Rapid, Multiplexed Molecular Assays. Front. Cell. Infect. Microbiol..

[51] ISBI Practice Guidelines Committee, Steering Subcommittee, Advisory Subcommittee (2016). ISBI Practice Guidelines for Burn Care. Burns.

[52] Huzar T.F., Cross J.M. (2011). Ventilator-Associated Pneumonia in Burn Patients: A Cause or Consequence of Critical Illness?. Expert Rev. Respir. Med..

[53] Barret J.P., Ramzy P.I., Wolf S.E., Herndon D.N. (1999). Sensitivity and Specificity of Bronchoalveolar Lavage and Protected Bronchial Brush in the Diagnosis of Pneumonia in Pediatric Burn Patients. Arch. Surg..

[54] Ramzy P.I., Herndon D.N., Wolf S.E., Irtun O., Barret J.P., Ramirez R.J., Heggers J.P. (1998). Comparison of Wound Culture and Bronchial Lavage in the Severely Burned Child: Implications for Antimicrobial Therapy. Arch. Surg..

[55] Church D., Elsayed S., Reid O., Winston B., Lindsay R. (2006). Burn Wound Infections. Clin. Microbiol. Rev..

[56] Rowan M.P., Cancio L.C., Elster E.A., Burmeister D.M., Rose L.F., Natesan S., Chan R.K., Christy R.J., Chung K.K. (2015). Burn Wound Healing and Treatment: Review and Advancements. Crit. Care.

[57] Azzopardi E.A., Azzopardi E., Camilleri L., Villapalos J., Boyce D.E., Dziewulski P., Dickson W.A., Whitaker I.S. (2014). Gram Negative Wound Infection in Hospitalised Adult Burn Patients--Systematic Review and Metanalysis-. PLoS ONE.

[58] Wasiak J., Cleland H., Campbell F., Spinks A. (2013). Dressings for Superficial and Partial Thickness Burns. Cochrane Database Syst. Rev..

[59] Brown T.P.L.H., Cancio L.C., McManus A.T., Mason A.D. (2004). Survival Benefit Conferred by Topical Antimicrobial Preparations in Burn Patients: A Historical Perspective. J. Trauma.

[60] Ruegsegger L., Xiao J., Naziripour A., Kanumuambidi T., Brown D., Williams F., Marshall S.H., Rudin S.D., Yen K., Chu T. (2022). Multidrug-Resistant Gram-Negative Bacteria in Burn Patients. Antimicrob. Agents Chemother..

[61] Gang R.K., Sanyal S.C., Bang R.L., Mokaddas E., Lari A.R. (2000). Staphylococcal Septicaemia in Burns. Burns.

[62] Altoparlak U., Erol S., Akcay M.N., Celebi F., Kadanali A. (2004). The Time-Related Changes of Antimicrobial Resistance Patterns and Predominant Bacterial Profiles of Burn Wounds and Body Flora of Burned Patients. Burns.

[63] Wisplinghoff H., Bischoff T., Tallent S.M., Seifert H., Wenzel R.P., Edmond M.B. (2004). Nosocomial Bloodstream Infections in US Hospitals: Analysis of 24,179 Cases from a Prospective Nationwide Surveillance Study. Clin. Infect. Dis..

[64] Suleyman G., Alangaden G.J. (2021). Nosocomial Fungal Infections: Epidemiology, Infection Control, and Prevention. Infect. Dis. Clin. N. Am..

[65] Ozlu O., Basaran A. (2022). Infections in Patients with Major Burns: A Retrospective Study of a Burn Intensive Care Unit. J. Burn Care Res..

[66] Emaneini M., Beigverdi R., van Leeuwen W.B., Rahdar H., Karami-Zarandi M., Hosseinkhani F., Jabalameli F. (2018). Prevalence of Methicillin-Resistant Staphylococcus Aureus Isolated from Burn Patients in Iran: A Systematic Review and Meta-Analysis. J. Glob. Antimicrob. Resist..

[67] Raz-Pasteur A., Fishel R., Hardak E., Mashiach T., Ullmann Y., Egozi D. (2016). Do Wound Cultures Give Information About the Microbiology of Blood Cultures in Severe Burn Patients?. Ann. Plast. Surg..

[68] Fransen F., Melchers M.J.B., Meletiadis J., Mouton J.W. (2016). Pharmacodynamics and Differential Activity of Nitrofurantoin against ESBL-Positive Pathogens Involved in Urinary Tract Infections. J. Antimicrob. Chemother..

[69] Costescu Strachinaru D.I., Gallez J.-L., François P.-M., Baekelandt D., Paridaens M.-S., Pirnay J.-P., De Vos D., Djebara S., Vanbrabant P., Strachinaru M. (2022). Epidemiology and Etiology of Blood Stream Infections in a Belgian Burn Wound Center. Acta Clin. Belg..

[70] Khalili Y., Memar M.Y., Farajnia S., Adibkia K., Kafil H.S., Ghotaslou R. (2021). Molecular Epidemiology and Carbapenem Resistance of *Pseudomonas aeruginosa* Isolated from Patients with Burns. J. Wound Care.

[71] Bahemia I.A., Muganza A., Moore R., Sahid F., Menezes C.N. (2015). Microbiology and Antibiotic Resistance in Severe Burns Patients: A 5 Year Review in an Adult Burns Unit. Burns.

[72] Girerd-Genessay I., Bénet T., Vanhems P. (2016). Multidrug-Resistant Bacterial Outbreaks in Burn Units: A Synthesis of the Literature According to the ORION Statement. J. Burn Care Res..

[73] Weber D.J., van Duin D., DiBiase L.M., Hultman C.S., Jones S.W., Lachiewicz A.M., Sickbert-Bennett E.E., Brooks R.H., Cairns B.A., Rutala W.A. (2014). Healthcare-Associated Infections among Patients in a Large Burn Intensive Care Unit: Incidence and Pathogens, 2008–2012. Infect. Control Hosp. Epidemiol..

[74] Rashid A., Solomon L.K., Lewis H.G., Khan K. (2006). Outbreak of Epidemic Methicillin-Resistant Staphylococcus Aureus in a Regional Burns Unit: Management and Implications. Burns.

[75] Falk P.S., Winnike J., Woodmansee C., Desai M., Mayhall C.G. (2000). Outbreak of Vancomycin-Resistant Enterococci in a Burn Unit. Infect. Control Hosp. Epidemiol..

[76] Kanamori H., Parobek C.M., Juliano J.J., van Duin D., Cairns B.A., Weber D.J., Rutala W.A. (2017). A Prolonged Outbreak of KPC-3-Producing Enterobacter Cloacae and Klebsiella Pneumoniae Driven by Multiple Mechanisms of Resistance Transmission at a Large Academic Burn Center. Antimicrob. Agents Chemother..

[77] Tissot F., Blanc D.S., Basset P., Zanetti G., Berger M.M., Que Y.-A., Eggimann P., Senn L. (2016). New Genotyping Method Discovers Sustained Nosocomial *Pseudomonas aeruginosa* Outbreak in an Intensive Care Burn Unit. J. Hosp. Infect..

[78] Douglas M.W., Mulholland K., Denyer V., Gottlieb T. (2001). Multi-Drug Resistant *Pseudomonas aeruginosa* Outbreak in a Burns Unit—An Infection Control Study. Burns.

[79] Simor A.E., Lee M., Vearncombe M., Jones-Paul L., Barry C., Gomez M., Fish J.S., Cartotto R.C., Palmer R., Louie M. (2002). An Outbreak Due to Multiresistant Acinetobacter Baumannii in a Burn Unit: Risk Factors for Acquisition and Management. Infect. Control Hosp. Epidemiol..

[80] Bassetti M., Rello J., Blasi F., Goossens H., Sotgiu G., Tavoschi L., Zasowski E.J., Arber M.R., McCool R., Patterson J.V. (2020). Systematic Review of the Impact of Appropriate versus Inappropriate Initial Antibiotic Therapy on Outcomes of Patients with Severe Bacterial Infections. Int. J. Antimicrob. Agents.

[81] Tang F., Yuan H., Li X., Qiao L. (2024). Effect of Delayed Antibiotic Use on Mortality Outcomes in Patients with Sepsis or Septic Shock: A Systematic Review and Meta-Analysis. Int. Immunopharmacol..

[82] Prescott H.C., Antonelli M., Alhazzanic W., Møller M.H., Alshamsi F., Azevedo L.C.P., Belley-Cote E., De Waele J., Derde L., Dionnec J.C. (2026). Surviving Sepsis Campaign: International Guidelines for Management of Sepsis and Septic Shock 2026. Intensive Care Med..

[83] Tamma P.D., Heil E.L., Justo J.A., Mathers A.J., Satlin M.J., Bonomo R.A. (2024). Infectious Diseases Society of America 2024 Guidance on the Treatment of Antimicrobial-Resistant Gram-Negative Infections. Clin. Infect. Dis..

[84] Paul M., Carrara E., Retamar P., Tängdén T., Bitterman R., Bonomo R.A., Waele J.d., Daikos G.L., Akova M., Harbarth S. (2022). European Society of Clinical Microbiology and Infectious Diseases (ESCMID) Guidelines for the Treatment of Infections Caused by Multidrug-Resistant Gram-Negative Bacilli (Endorsed by European Society of Intensive Care Medicine). Clin. Microbiol. Infect..

[85] Liu V.X., Fielding-Singh V., Greene J.D., Baker J.M., Iwashyna T.J., Bhattacharya J., Escobar G.J. (2017). The Timing of Early Antibiotics and Hospital Mortality in Sepsis. Am. J. Respir. Crit. Care Med..

[86] Ferrer R., Martin-Loeches I., Phillips G., Osborn T.M., Townsend S., Dellinger R.P., Artigas A., Schorr C., Levy M.M. (2014). Empiric Antibiotic Treatment Reduces Mortality in Severe Sepsis and Septic Shock from the First Hour: Results from a Guideline-Based Performance Improvement Program. Crit. Care Med..

[87] Raith E.P., Udy A.A., Bailey M., McGloughlin S., MacIsaac C., Bellomo R., Pilcher D.V., Australian and New Zealand Intensive Care Society (ANZICS) Centre for Outcomes and Resource Evaluation (CORE) (2017). Prognostic Accuracy of the SOFA Score, SIRS Criteria, and qSOFA Score for In-Hospital Mortality Among Adults with Suspected Infection Admitted to the Intensive Care Unit. JAMA.

[88] Vincent J.L., Moreno R., Takala J., Willatts S., De Mendonça A., Bruining H., Reinhart C.K., Suter P.M., Thijs L.G. (1996). The SOFA (Sepsis-Related Organ Failure Assessment) Score to Describe Organ Dysfunction/Failure. On Behalf of the Working Group on Sepsis-Related Problems of the European Society of Intensive Care Medicine. Intensive Care Med..

[89] NHS National Early Warning Score (NEWS) 2 Standardising the Assessment of Acute-Illness Severity in the NHS 2017. https://www.rcp.ac.uk/media/a4ibkkbf/news2-final-report_0_0.pdf.

[90] Ladhani H.A., Sajankila N., Zosa B.M., He J.C., Yowler C.J., Brandt C., Claridge J.A., Khandelwal A.K. (2018). Utility of Sequential Organ Failure Assessment Score in Predicting Bacteremia in Critically Ill Burn Patients. Am. J. Surg..

[91] Yoon J., Kym D., Hur J., Cho Y.S., Chun W., Yoon D. (2023). Validation of Sepsis-3 Using Survival Analysis and Clinical Evaluation of Quick SOFA, SIRS, and Burn-Specific SIRS for Sepsis in Burn Patients with Suspected Infection. PLoS ONE.

[92] Gimenez F.M.P., Cardoso L.T.Q., Kerbauy G., Matsuo T., Grion C.M.C. (2025). Analysis of the SOFA Score, Quick-SOFA, and SIRS Criteria in Burn Patients with Infection. Rev. Bras. Enferm..

[93] Osler T., Glance L.G., Hosmer D.W. (2010). Simplified Estimates of the Probability of Death after Burn Injuries: Extending and Updating the Baux Score. J. Trauma.

[94] Knowlin L., Stanford L., Moore D., Cairns B., Charles A. (2016). The Measured Effect Magnitude of Co-Morbidities on Burn Injury Mortality. Burns.

[95] Heng J.S., Clancy O., Atkins J., Leon-Villapalos J., Williams A.J., Keays R., Hayes M., Takata M., Jones I., Vizcaychipi M.P. (2015). Revised Baux Score and Updated Charlson Comorbidity Index Are Independently Associated with Mortality in Burns Intensive Care Patients. Burns.

[96] Prasad A., Thode H.C., Singer A.J. (2020). Predictive Value of Quick SOFA and Revised Baux Scores in Burn Patients. Burns.

[97] Gallardo-Pizarro A., Lopera C., Peyrony O., Monzo-Gallo P., Aiello T.F., Martinez-Urrea A., Herrera S., Río A.D., Teijon-Lumbreras C., Chumbita M. (2025). Rectal Colonisation by Multidrug-Resistant Gram-Negative Bacteria and Subsequent Bacteraemia in Haematological Patients. Clin. Microbiol. Infect..

[98] Temkin E., Solter E., Lugassy C., Chen D., Cohen A., Schwaber M.J., Carmeli Y. (2024). CPE Working Group The Natural History of Carbapenemase-Producing Enterobacterales: Progression From Carriage of Various Carbapenemases to Bloodstream Infection. Clin. Infect. Dis..

[99] Giannella M., Pascale R., Viale P. (2024). Progression From Carriage to Bloodstream Infection and Fatality by Different Enterobacterales Species, Carbapenemases, and Host Settings: Deciphering the Melting Pot. Clin. Infect. Dis..

[100] Kang S.-W., Park S., Kim A.R., Han J., Lee J., Seo H., Sung H., Kim M.-N., Chang E., Bae S. (2023). Clinical Characteristics of and Risk Factors for Subsequent Carbapenemase-Producing Enterobacterales (CPE) Bacteraemia in Rectal CPE Carriers. Int. J. Antimicrob. Agents.

[101] Cano A., Gutiérrez-Gutiérrez B., Machuca I., Gracia-Ahufinger I., Pérez-Nadales E., Causse M., Castón J.J., Guzman-Puche J., Torre-Giménez J., Kindelán L. (2018). Risks of Infection and Mortality Among Patients Colonized With Klebsiella Pneumoniae Carbapenemase-Producing K. Pneumoniae: Validation of Scores and Proposal for Management. Clin. Infect. Dis..

[102] Kim M., Jeon K., Kym D., Jung J., Jang Y.J., Han S.B. (2025). Carbapenem-Resistant *Enterobacterales* Infection and Colonization in Patients with Severe Burns: A Retrospective Cohort Study in a Single Burn Center. Antimicrob. Resist. Infect. Control.

[103] Reighard A., Diekema D., Wibbenmeyer L., Ward M., Herwaldt L. (2009). Staphylococcus Aureus Nasal Colonization and Colonization or Infection at Other Body Sites in Patients on a Burn Trauma Unit. Infect. Control Hosp. Epidemiol..

[104] Gardner A., Nieberg P., Sakoulas G., Wong-Beringer A. (2025). Carbapenem De-Escalation as an Antimicrobial Stewardship Strategy: A Narrative Review. JAC Antimicrob. Resist..

[105] Mathieu C., Pastene B., Cassir N., Martin-Loeches I., Leone M. (2019). Efficacy and Safety of Antimicrobial De-Escalation as a Clinical Strategy. Expert Rev. Anti-Infect. Ther..

[106] Kohama Y., Kosugi M., Arakawa M., Hidaka S. (2022). Evaluating the Impact of De-Escalating Antimicrobial Therapy in Burn Patients: A Retrospective Cohort Study. Pharmazie.

[107] Averbuch D., Vanbiervliet Y., Baccelli F., Mikulska M., Neofytos D., Garcia-Vidal C., Aguilar-Guisado M., Blijlevens N., Munoz P., Cordonnier C. (2026). Empirical and Targeted Antimicrobial Therapy in Patients with Febrile Neutropenia and Haematological Malignancy or after Haematopoietic Cell Transplantation: Recommendations from the 10th European Conference on Infections in Leukaemia. Lancet Infect. Dis..

[108] Silverman J.A., Mortin L.I., Vanpraagh A.D.G., Li T., Alder J. (2005). Inhibition of Daptomycin by Pulmonary Surfactant: In Vitro Modeling and Clinical Impact. J. Infect. Dis..

[109] Burdet C., Saïdani N., Dupieux C., Lemaignen A., Canouï E., Surgers L., Vareil M.O., Lefort A., Lepeule R., Peiffer-Smadja N. (2025). Cloxacillin versus Cefazolin for Meticillin-Susceptible Staphylococcus Aureus Bacteraemia (CloCeBa): A Prospective, Open-Label, Multicentre, Non-Inferiority, Randomised Clinical Trial. Lancet.

[110] Lakhundi S., Zhang K. (2018). Methicillin-Resistant Staphylococcus Aureus: Molecular Characterization, Evolution, and Epidemiology. Clin. Microbiol. Rev..

[111] Blanchet B., Jullien V., Vinsonneau C., Tod M. (2008). Influence of Burns on Pharmacokinetics and Pharmacodynamics of Drugs Used in the Care of Burn Patients. Clin. Pharmacokinet..

[112] Cota J.M., FakhriRavari A., Rowan M.P., Chung K.K., Murray C.K., Akers K.S. (2016). Intravenous Antibiotic and Antifungal Agent Pharmacokinetic-Pharmacodynamic Dosing in Adults with Severe Burn Injury. Clin. Ther..

[113] Jeschke M.G. (2016). Postburn Hypermetabolism: Past, Present, and Future. J. Burn Care Res..

[114] Steele A.N., Grimsrud K.N., Sen S., Palmieri T.L., Greenhalgh D.G., Tran N.K. (2015). Gap Analysis of Pharmacokinetics and Pharmacodynamics in Burn Patients: A Review. J. Burn Care Res..

[115] Abdul-Aziz M.H., Alffenaar J.-W.C., Bassetti M., Bracht H., Dimopoulos G., Marriott D., Neely M.N., Paiva J.-A., Pea F., Sjovall F. (2020). Antimicrobial Therapeutic Drug Monitoring in Critically Ill Adult Patients: A Position Paper. Intensive Care Med..

[116] Gatti M., Cojutti P.G., Pascale R., Tonetti T., Laici C., Dell’Olio A., Siniscalchi A., Giannella M., Viale P., Pea F. (2021). Assessment of a PK/PD Target of Continuous Infusion Beta-Lactams Useful for Preventing Microbiological Failure and/or Resistance Development in Critically Ill Patients Affected by Documented Gram-Negative Infections. Antibiotics.

[117] Roberts J.A., Paul S.K., Akova M., Bassetti M., Waele J.J.D., Dimopoulos G., Kaukonen K.-M., Koulenti D., Martin C., Montravers P. (2014). DALI: Defining Antibiotic Levels in Intensive Care Unit Patients: Are Current β-Lactam Antibiotic Doses Sufficient for Critically Ill Patients?. Clin. Infect. Dis..

[118] Conil J.-M., Georges B., Ravat F., Ruiz S., Seguin T., Metsu D., Fourcade O., Saivin S. (2013). Ceftazidime Dosage Recommendations in Burn Patients: From a Population Pharmacokinetic Approach to Clinical Practice via Monte Carlo Simulations. Clin. Ther..

[119] Dailly E., Kergueris M.F., Pannier M., Jolliet P., Bourin M. (2003). Population Pharmacokinetics of Imipenem in Burn Patients. Fundam. Clin. Pharmacol..

[120] Doh K., Woo H., Hur J., Yim H., Kim J., Chae H., Han S., Yim D.-S. (2010). Population Pharmacokinetics of Meropenem in Burn Patients. J. Antimicrob. Chemother..

[121] Machado A.S., Oliveira M.S., Sanches C., da Silva Junior C.V., Gomez D.S., Gemperli R., Santos S.R.C.J., Levin A.S. (2017). Clinical Outcome and Antimicrobial Therapeutic Drug Monitoring for the Treatment of Infections in Acute Burn Patients. Clin. Ther..

[122] Falcone M., Menichetti F., Cattaneo D., Tiseo G., Baldelli S., Galfo V., Leonildi A., Tagliaferri E., Di Paolo A., Pai M.P. (2021). Pragmatic Options for Dose Optimization of Ceftazidime/Avibactam with Aztreonam in Complex Patients. J. Antimicrob. Chemother..

[123] Walstad R.A., Aanderud L., Thurmann-Nielsen E. (1988). Pharmacokinetics and Tissue Concentrations of Ceftazidime in Burn Patients. Eur. J. Clin. Pharmacol..

[124] Fournier A., Eggimann P., Pantet O., Pagani J.L., Dupuis-Lozeron E., Pannatier A., Sadeghipour F., Voirol P., Que Y.-A. (2018). Impact of Real-Time Therapeutic Drug Monitoring on the Prescription of Antibiotics in Burn Patients Requiring Admission to the Intensive Care Unit. Antimicrob. Agents Chemother..

[125] Alshaer M., Mazirka P., Burch G., Peloquin C., Drabick Z., Carson J. (2023). Experience with Implementing a Beta-Lactam Therapeutic Drug Monitoring Service in a Burn Intensive Care Unit: A Retrospective Chart Review. J. Burn Care Res..

[126] Santos R.M., Boyd A.N., Walroth T.A., Hall A., King J., Ahiskali A., Walter E., Neumann N., Curry D., Hoyte B. (2024). A Multicenter, Retrospective Outcome Analysis of Vancomycin Area Under the Curve Versus Trough-Based Dosing Strategies in Patients with Burn or Inhalational Injuries (MONITOR). J. Burn Care Res..

[127] Bousselmi K., Thabet L., Bayoudh A., Memi M., Messaadi A. (2006). Antimicrobial Use and Antimicrobial Resistance in an Intensive Care Burn Department. Crit. Care.

[128] Wermine K., Gotewal S., Song J., Huang L.G., Corona K.K., Chokshi S.N., Villarreal E.L., Efejuku T.A., Chaij J.M., Bagby S.P. (2024). Patterns of Antibiotic Administration in Patients with Burn Injuries: A TriNetX Study. Burns.

[129] Dyar O.J., Huttner B., Schouten J., Pulcini C., ESGAP (ESCMID Study Group for Antimicrobial stewardshiP) (2017). What Is Antimicrobial Stewardship?. Clin. Microbiol. Infect..

[130] Sehgal P., Elligsen M., Lo J., Lam P.W., Leis J.A., Fowler R., Pinto R., Daneman N. (2021). Long-Term Sustainability and Acceptance of Antimicrobial Stewardship in Intensive Care: A Retrospective Cohort Study. Crit. Care Med..

[131] Pulcini C., Tebano G., Mutters N.T., Tacconelli E., Cambau E., Kahlmeter G., Jarlier V., EUCIC-ESGAP-EUCAST Selective Reporting Working Group (2017). Selective Reporting of Antibiotic Susceptibility Test Results in European Countries: An ESCMID Cross-Sectional Survey. Int. J. Antimicrob. Agents.

[132] Kaki R., Elligsen M., Walker S., Simor A., Palmay L., Daneman N. (2011). Impact of Antimicrobial Stewardship in Critical Care: A Systematic Review. J. Antimicrob. Chemother..

[133] Guo Y., Kong L., Liu J., Li R., Cui M., Kong X., Li X., Yang M. (2025). Evaluation of an Antibiotic Stewardship Program for Promoting Rational Antibiotic Use in an ICU in China. BMC Infect. Dis..

[134] Schuetz P., Beishuizen A., Broyles M., Ferrer R., Gavazzi G., Gluck E.H., González Del Castillo J., Jensen J.-U., Kanizsai P.L., Kwa A.L.H. (2019). Procalcitonin (PCT)-Guided Antibiotic Stewardship: An International Experts Consensus on Optimized Clinical Use. Clin. Chem. Lab. Med..

[135] Wirz Y., Meier M.A., Bouadma L., Luyt C.E., Wolff M., Chastre J., Tubach F., Schroeder S., Nobre V., Annane D. (2018). Effect of Procalcitonin-Guided Antibiotic Treatment on Clinical Outcomes in Intensive Care Unit Patients with Infection and Sepsis Patients: A Patient-Level Meta-Analysis of Randomized Trials. Crit. Care.

[136] Matuszak S.S., Kolodziej L., Micek S., Kollef M. (2025). Antibiotic De-Escalation in the Intensive Care Unit: Rationale and Potential Strategies. Antibiotics.

[137] Chiotos K., Tamma P.D., Gerber J.S. (2019). Antibiotic Stewardship in the Intensive Care Unit: Challenges and Opportunities. Infect. Control Hosp. Epidemiol..

[138] Pickens C.I., Wunderink R.G. (2019). Principles and Practice of Antibiotic Stewardship in the ICU. Chest.

[139] Burgoon R., Weeda E., Mediwala K.N., Raux B.R. (2022). Clinical Utility of Negative Methicillin-Resistant Staphylococcus Aureus (MRSA) Nasal Surveillance Swabs in Skin and Skin Structure Infections. Am. J. Infect. Control.

[140] Karlin D., Pham C., Furukawa D., Kaur I., Martin E., Kates O., Vijayan T. (2024). State-of-the-Art Review: Use of Antimicrobials at the End of Life. Clin. Infect. Dis..

[141] Barajas-Nava L.A., López-Alcalde J., Figuls M.R.I., Solà I., Cosp X.B. (2013). Antibiotic Prophylaxis for Preventing Burn Wound Infection. Cochrane Database Syst. Rev..

[142] Avni T., Levcovich A., Ad-El D.D., Leibovici L., Paul M. (2010). Prophylactic Antibiotics for Burns Patients: Systematic Review and Meta-Analysis. BMJ.

[143] Ramos G., Cornistein W., Cerino G.T., Nacif G. (2017). Systemic Antimicrobial Prophylaxis in Burn Patients: Systematic Review. J. Hosp. Infect..

[144] Zha S., Niu J., He Z., Fu W., Huang Q., Guan L., Zhou L., Chen R. (2023). Prophylactic Antibiotics for Preventing Ventilator-Associated Pneumonia: A Pairwise and Bayesian Network Meta-Analysis. Eur. J. Med. Res..

[145] Evans L., Rhodes A., Alhazzani W., Antonelli M., Coopersmith C.M., French C., Machado F.R., Mcintyre L., Ostermann M., Prescott H.C. (2021). Surviving Sepsis Campaign: International Guidelines for Management of Sepsis and Septic Shock 2021. Crit. Care Med..

[146] Daneman N., Rishu A., Pinto R., Rogers B.A., Shehabi Y., Parke R., Cook D., Arabi Y., Muscedere J., Reynolds S. (2025). Antibiotic Treatment for 7 versus 14 Days in Patients with Bloodstream Infections. N. Engl. J. Med..

[147] Chastre J., Wolff M., Fagon J.-Y., Chevret S., Thomas F., Wermert D., Clementi E., Gonzalez J., Jusserand D., Asfar P. (2003). Comparison of 8 vs. 15 Days of Antibiotic Therapy for Ventilator-Associated Pneumonia in Adults: A Randomized Trial. JAMA.

[148] Mo Y., Booraphun S., Li A.Y., Domthong P., Kayastha G., Lau Y.H., Chetchotisakd P., Limmathurotsakul D., Tambyah P.A., Cooper B.S. (2024). Individualised, Short-Course Antibiotic Treatment versus Usual Long-Course Treatment for Ventilator-Associated Pneumonia (REGARD-VAP): A Multicentre, Individually Randomised, Open-Label, Non-Inferiority Trial. Lancet Respir. Med..

[149] Lee T.C., Prosty C.J., Fralick M., Huttner A., McDonald E.G., Molina J., Paul M., Pinto R., Rishu A., von Dach E. (2025). Seven vs Fourteen Days of Antibiotics for Gram-Negative Bloodstream Infection: A Systematic Review and Noninferiority Meta-Analysis. JAMA Netw. Open.

[150] Nauriyal V., Rai S.M., Joshi R.D., Thapa B.B., Kaljee L., Prentiss T., Maki G., Shrestha B., Bajracharya D.C., Karki K. (2020). Evaluation of an Antimicrobial Stewardship Program for Wound and Burn Care in Three Hospitals in Nepal. Antibiotics.

[151] Fonseca-Rivera I.C., Pando-Caciano A., Agüero-Palacios Y.D. (2025). Impact of an Antimicrobial Stewardship Program on Mortality and Consumption of Antibiotics in the Intensive Care Units of a Pediatric Referral Hospital in Peru. Antimicrob. Steward. Healthc. Epidemiol..

[152] Sadeq A.A., Shamseddine J.M., Babiker Z.O.E., Nsutebu E.F., Moukarzel M.B., Conway B.R., Hasan S.S., Conlon-Bingham G.M., Aldeyab M.A. (2021). Impact of Multidisciplinary Team Escalating Approach on Antibiotic Stewardship in the United Arab Emirates. Antibiotics.

[153] Zbyrak V., Reverón S.L., Smoke S., Mehta A., Marano M.A., Lee R. (2020). Antibiotic Usage After Procalcitonin-Guided Therapy Algorithm Implementation In A Burn Intensive Care Unit. Ann. Burn Fire Disasters.

